# Noninvasive transcranial classification of stroke using a portable eddy current damping sensor

**DOI:** 10.1038/s41598-021-89735-x

**Published:** 2021-05-13

**Authors:** Shane Shahrestani, Gabriel Zada, Tzu-Chieh Chou, Brandon Toy, Bryan Yao, Norman Garrett, Nerses Sanossian, Andrew Brunswick, Kuang-Ming Shang, Yu-Chong Tai

**Affiliations:** 1 Department of Medical Engineering, California Institute of Technology, Pasadena, CA USA; 2 Department of Neurosurgery, Keck School of Medicine, University of Southern California, Los Angeles, CA USA; 3 Department of Neurology, Keck School of Medicine, University of Southern California, Los Angeles, CA USA

**Keywords:** Neurological disorders, Cerebrovascular disorders, Stroke, Translational research

## Abstract

Existing paradigms for stroke diagnosis typically involve computed tomography (CT) imaging to classify ischemic versus hemorrhagic stroke variants, as treatment for these subtypes varies widely. Delays in diagnosis and transport of unstable patients may worsen neurological status. To address these issues, we describe the development of a rapid, portable, and accurate eddy current damping (ECD) stroke sensor. Copper wire was wound to create large (11.4 cm), medium (4.5 cm), and small (1.5 cm) solenoid coils with varying diameters, with each connected to an inductance-to-digital converter. Eight human participants were recruited between December 15, 2019 and March 15, 2020, including two hemorrhagic stroke, two ischemic stroke, one subarachnoid hemorrhage, and three control participants. Observers were blinded to lesion type and location. A head cap with 8 horizontal scanning paths was placed on the patient. The sensor was tangentially rotated across each row on the patient’s head circumferentially. Consent, positioning, and scanning with the sensor took roughly 15 min from start to end for each participant and all scanning took place at the patient bedside. The ECD sensor accurately classified and imaged each of the varying stroke types in each patient. The sensor additionally detected ischemic and hemorrhagic lesions located deep inside the brain, and its range is selectively tunable during sensor design and fabrication.

## Introduction

Stroke can be classified as either ischemic or hemorrhagic, and stroke misclassification and mistreatment can easily lead to disease exacerbation, worsened neurological status, and/or mortality. The mainstay for ischemic stroke when detected within a certain time window is administration of tissue plasminogen activator (tPA) in an attempt to dissolve the clot. On the other hand, in patients with hemorrhagic stroke (also known as an intracerebral hematoma or ICH), tPA is contraindicated, and guidelines suggest supportive therapy, blood pressure control, and selective neurosurgical evacuation^1^. Thus, diagnostic computed tomography (CT) imaging is typically performed before treatment to classify the stroke by ischemic versus hemorrhagic subtypes^1,2^. As a result, the time lapse between ictus, field intervention, hospital transport, and treatment often exceeds 2 h, with most of this time being consumed by waiting for diagnostic imaging to be performed and awaiting interpretation of scan results^2–4^. In stroke management, time to treatment is directly proportional to patient morbidity and mortality, due to the progressive death of ischemic neurons. The pharmacodynamics of ischemic stroke medications such as tPA typically mandate treatment within 4.5 h of the onset of ischemic stroke to minimize adverse drug-related events^5,6^. With stroke affecting roughly 800,000 people in the US per year, and hovering at the fifth highest cause of death, rapid field or bedside stroke diagnosis and treatment may be made more efficient in an effort to minimize stroke morbidity and mortality^7^.

We demonstrate stroke detection and classification using a novel eddy current damping (ECD) sensor. Different organs in the body have varying electrical conductivities, and these values are highly dependent on cellular composition. The brain contains a wide range of conductivities, the lowest being found in the myelin-predominant parenchyma (0.2 S/m), with unclotted and clotted blood having higher conductivity values (0.65 S/m) and ischemic tissue being half as conductive as normal brain parenchyma (0.1 S/m)^8–11^. Thus, we set out to develop a sensor capable of sensing regions with abnormally increased or decreased conductivities to provide first responders and emergency room physicians with information to efficiently guide subsequent clinical treatment decisions.

## Methods

### Sensor principles

Our equivalent circuit model consists of a sensor coil paired with a capacitor to form an electrical resonant circuit operating at 1 MHz, which is significantly different from the architecture of previous ECD sensors used in industry for metal detection and aerospace for crack inspection consisting of a bridge circuit that measures the sensor coil impedance^12,13^. The sensor operates as a coil carrying an alternating current (AC), which produces a time-varying magnetic field. Magnetic fields generated by the inductor produce an electromotive force (EMF) that creates a looping ‘eddy’ current in the conductive material described through Ohm’s law.

As a result, when a conductive target, such as a blood/haemorrhage, is placed within range of the sensor, eddy currents generated within the target produce a counteracting magnetic field resulting in a decrease in coil inductance and rise in coil resonant frequency. Conversely, when a less conductive target, such as an infarct, is brought within range of the sensor, coil inductance increases, and coil resonant frequency decreases. These electromagnetic variations can be detected within our circuit using a frequency counter, producing unique signatures for both hemorrhage and ischemia. At the same time, the EMF generated by the counteracting magnetic field impedes current flow within the coil and increases the coil’s AC resistance. Because the parallel resistance (R_p_) of the electrical resonant circuit is inversely related to the coil’s AC resistance, it is possible to calculate unique R_p_ values when conductive materials are introduced. We use the Texas Instruments LDC 1101 chip to convert signals from our coils into computer readout^14^. Each coil is connected to its own chip, with readout sent to a laptop in series.

### Sensor construction

**Figure 1:**
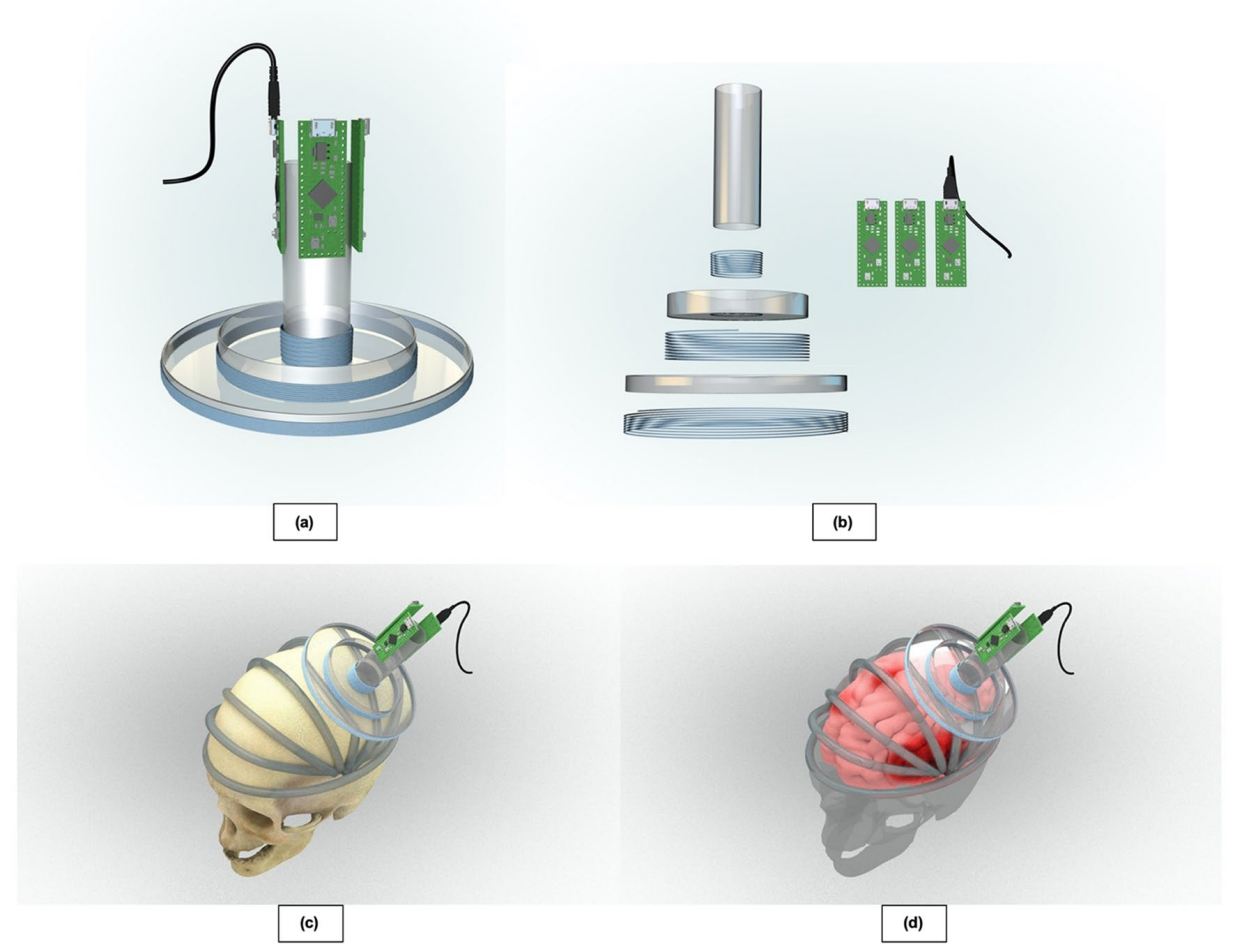
(**a)** 3D models of the three coil ECD sensor is shown. The variability in coil sizes allows for scanning at different depths, and the concentric design aligns the magnetic field distributions of each sensor to increase scanning specificity. In image (**b**), we take the ECD sensors apart to demonstrate their components, which include three wire solenoids, three plastic frames, and three inductance-to-digital converting devices. (**c**) The sensor is moved across the eight scanning paths while recording R_p_ values. (**d**) Areas of bleeding result in decreased R_p_ measurements, while areas of normal brain tissue leave R_p_ unchanged.

In our experiment, we constructed three solenoid coils with varying diameters and number of turns out of Litz wire, with the largest coil having an outer diameter of 11.4 cm and 6 turns, the middle having an outer diameter of 4.5 cm and 15 turns, and the smallest having an outer diameter of 1.5 cm and 35 turns (Fig. 1). The largest coil was used for localization and imaging, and the medium and small coils were used for location confirmation and to quantify the depth of the bleed.

### Finite element model

COMSOL multiphysics modelling software (Version 5.5, COMSOL Inc., Burlington, MA) was utilized to analyze the magnetic field space of the ECD sensor. We generated both three-dimensional (3D) and two-dimensional (2D) models of the human head with simulated lesion. All simulations were made from homogenized models, which allowed for reduction of computational effort without compromising the accuracy of the results. Text files of coordinate data from publicly available MRI head scanning were imported as an interpolation curve and organized in sectionwise format and lofting of the closed curve allowed for the generation of a solid object^15,16^.

### Live human experiments

Institutional Review Board (IRB) approval was obtained at the Keck School of Medicine of USC to conduct a prospective, nonrandomized, blinded proof-of-concept study to assess the efficacy of this sensor (IRB Number HS-19-00714) between December 15, 2019 and March 15, 2020. Informed consent was obtained by a neurosurgeon investigator (GZ) from all patients prior to testing. All methods were carried out in accordance with relevant guidelines and regulations. Hemorrhagic stroke patients (n = 3), ischemic stroke patients (n = 2), and healthy control patients without a stroke (n = 3) were enrolled for this study. Data obtained from participants in the control group provided baseline R_p_ values for each area of the brain and was used for differential imaging during image production. As such, when data was obtained from patients, our algorithm compared the resistance values to those of healthy patients to identify and classify potential lesions. Patients experiencing stroke symptoms were admitted and provided workup according to the traditional standard of care. CT imaging was used to determine the location, depth, volume and type of injury in accordance to the normal standard of care. Immediately after imaging, the sensor was used to collect data in a blinded fashion, and results were compared to CT imaging. We ensured that no metallic objects were in the range of detection of the coils. A head cap with 8 equidistant tubes was placed such that the most anterior tube crossed the forehead horizontally, and the most posterior tube crossed the nape of the neck horizontally. This horizontal alignment allowed for appropriate spatial coverage of the participant’s head, and each horizontal tube corresponded to a horizontal row on the heatmap, allowing for image reconstruction of the lesion with reference to anatomical landmarks. All scanning on stroke patients was done at the patient’s bedside.

### Image production

Data from the sensors was filtered using Savitzky-Golay smoothing to remove high frequency noise, the continuously-collected data from each row was down-sampled from ≥ 40,000 data points to eight averaged and equidistant points, and control values were subtracted to produce a differential signal. We use the eight averaged data points from the eight scanning rows to create an 8 × 8 conductivity heatmap, with brighter areas indicating higher probability of hemorrhage locations. Lesions, if present, were shown on the vertices of the 8 × 8 grid and interpolation was performed within each heatmap to estimate the margins of the lesion. Colors were inverted if an ischemic stroke was suspected, and imaging showed a bright background with dark areas indicating higher probability of ischemia. This image was then mapped to the curved surface of a 3D hemisphere, with the top and bottom edges of the heatmap mapped to the front and back half-edges of the hemisphere (front and back of the head), and the left and right edges of the heatmap mapped to singular points on the left and right sides of the hemisphere (above each ear). A board-certified neuroradiologist (NS) was also recruited to confirm the accuracy of the sensor output.

## Results

### Sensor analysis

**Figure 2:**
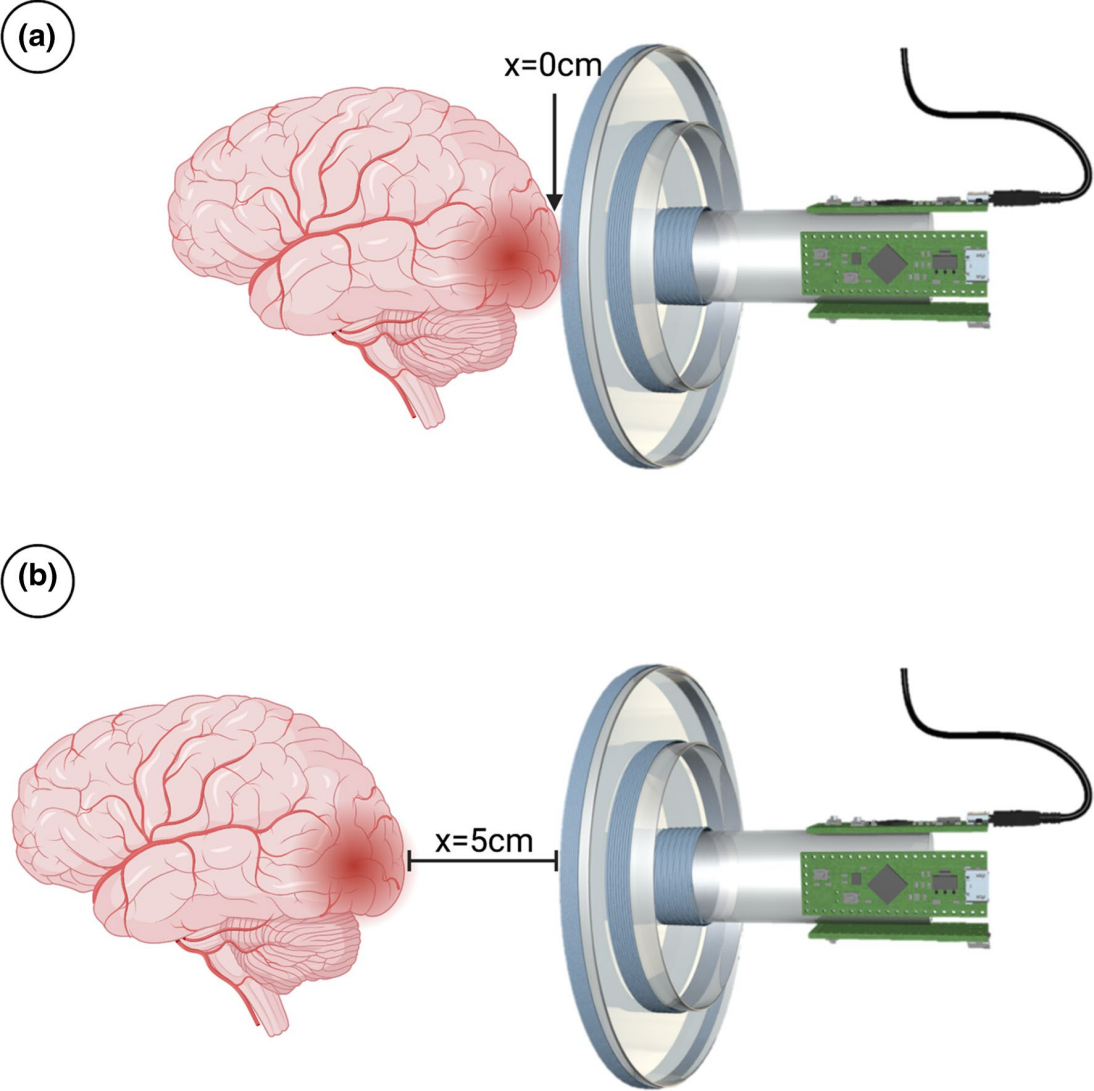
Schematic of the sensor and brain lesion at a distance of (**a**) x = 0 cm and (**b**) x = 5 cm. These distances from the center of the coil along the axis of symmetry are utilized to calculate the magnitude of the magnetic field using Biot-Savart’s Law. Image created using Biorender.com.

Using Biot-Savart’s law, the expected magnetic field strength of each coil along the axis of symmetry of the sensor was calculated. For the largest coil created thus far (11.4 cm diameter), the maximum magnetic field flux densities at the sensor (x = 0, Fig. 2a), 2.5 cm away from the sensor (x = 2.5), and 5 cm away from the sensor (x = 5, Fig. 2b) are 0.1786 μT, 0.1372 μT, and 0.0759 μT respectively. For the medium coil (4.5 cm), the maximum magnetic field flux densities at the sensor (x = 0), 2.5 cm away from the sensor (x = 2.5), and 5 cm away from the sensor (x = 5) are 1.131 μT, 0.3386 μT, 0.0782 μT respectively. For the smallest coil (1.5 cm diameter) the maximum magnetic field flux densities at the sensor (x = 0), 2.5 cm away from the sensor (x = 2.5), and 5 cm away from the sensor (x = 5) are 7.917 μT, 0.1878 μT, and 0.0258 μT respectively. Full equations may be seen in Supplementary Equations. COMSOL multiphysics simulation for each coil revealed that the predicted magnetic field flux densities for the large, medium, and small coils at a distance of x = 0 were 0.1335 μT, 0.6295 μT, and 3.492 μT respectively, which also followed the trend of experimental values. COMSOL multiphysics simulation for each coil revealed that the predicted magnetic field flux densities for the large, medium, and small coils at 5 cm away from the sensor (x = 5) were 0.0567 μT, 0.0435 μT, and 0.0114 μT respectively which were comparable to the flux densities obtained experimentally from Biot-Savart’s Law. These magnetic field calculations inform that: (1) The magnetic fields created by ECD sensors pose no safety concern to patients and coil operators and (2) smaller coils have a greater sensing ability closer to the coil and their magnetic fields decay quickly as a target is moved away, and larger coils have a weaker but more consistent sensing ability as the target is moved away. By varying the diameter, turn number, and length of the coil it is possible to design several sensors with varying detection depths. Multiple coils can be assembled concentrically to create a single sensor.

### Tuning curves

**Figure 3:**
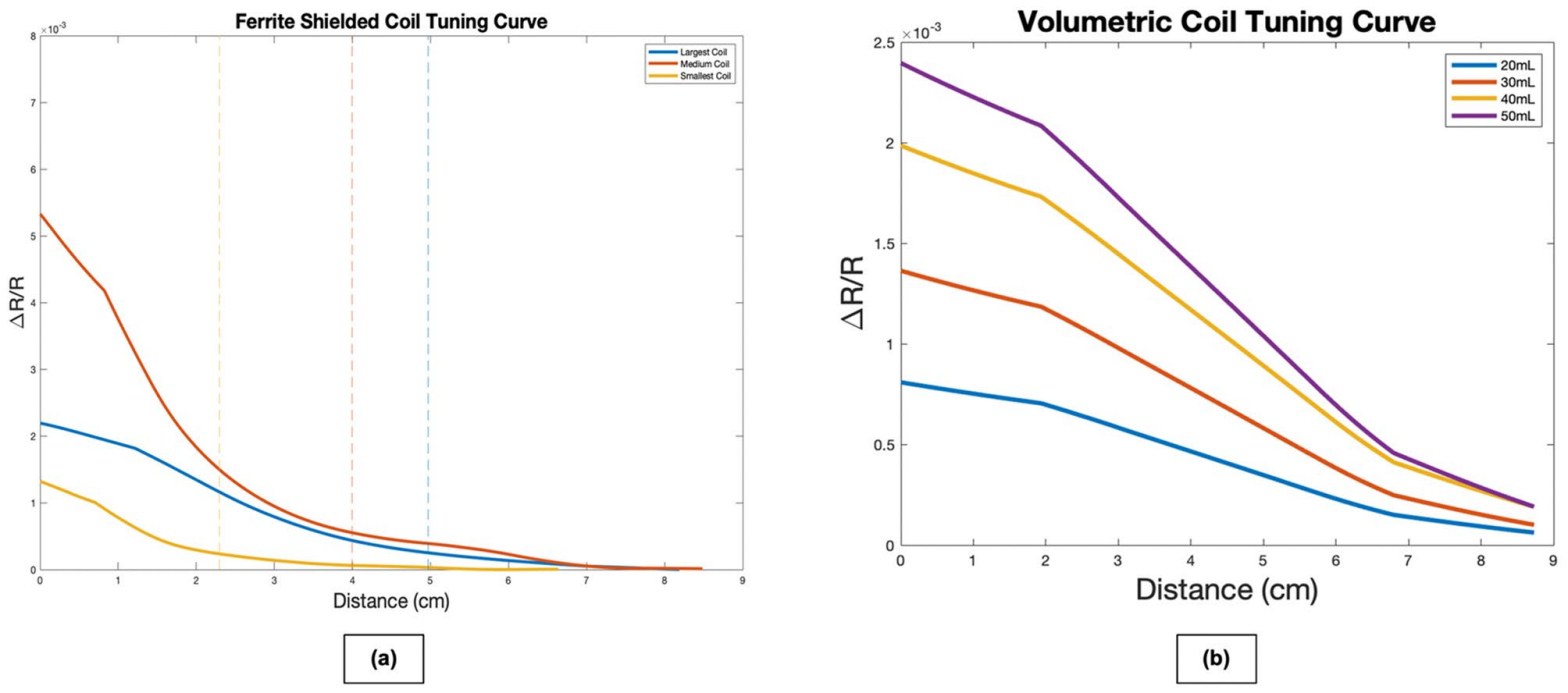
(**a**) The curves were produced by moving a 5 cm spherical balloon towards each sensor at a constant rate, starting at a distance of 8 cm and moving closer until the balloon and sensor were touching (0 cm). Maximum sensor range was defined as a 10% change in resistance from baseline ($\frac{\mathrm{\Delta R}}{\mathrm{R}}$=0), which was determined to be the chance in signal necessary to overcome noise (SNR = 10). At the point where the signal is 10% of the maximum generated signal, we draw a vertical line and call it the distance threshold (the point at which we have overcome noise, as defined by the SNR, and we can be confident we are detecting changes in signal). The distance threshold is 4.97 cm for the large coil (blue, 3.99 cm for the medium coil (red), and 2.29 cm for the small coil (yellow). As expected, maximum sensor range varied directly as a function of coil size, with the largest coil having the largest range and the smallest coil having the smallest range. While the medium coil rises much faster than the large coil, the 10% threshold is lower, as expected, than the large coil. Using these ranges, we can predict lesion depth based on the pattern of activated sensors. (**b**) Tuning curves showing the change in resistance as a function of volume in a solenoid coil, demonstrating a volumetric dose–response relationship. These curves may be used to predict the volume of lesion.

Spherical saline-filled balloons of different sizes were moved at a constant rate towards the sensors. A signal was detectable with the threshold signal being defined as 10% of the change from baseline to endpoint. The 10% value was obtained from the sensor signal-to-noise ratio (SNR), which was found to be 10. As such, a 10% change would be above the noise threshold and accurately indicate detection of a lesion. Since increasing coil sizes and turn numbers intrinsically correlate with increasing magnetic field strength, we created tuning curves for each coil (Fig. 3a). The tuning curve demonstrates that the large coil has the largest range of detection (4.97 cm), followed by the medium sized coil (3.99 cm), and lastly the small coil (2.29 cm). Furthermore, balloons of different sizes were used to create volume-dependent tuning curves (Fig. 3b). As seen in these curves, the sensors have a reproducible volume-dependent signal change, with larger and more conductive lesions eliciting an increased resistance change, and smaller and less conductive lesions eliciting a decreased resistance change. Thus, these tuning curves can be utilized to approximate the depth and volume of a signal-producing lesion by understanding which coils detect a signal in series coil measurements. It then follows that a multi-coiled sensor design may not only be able to localize lesions, but it also may provide important clinical information pertaining to the depth and volume of the lesion within the brain.

### Live human experiments

A wearable head cap with eight equidistant scanning paths was developed for live human testing. The entire process—which included obtaining consent, positioning each patient, and scanning with the sensors—took 15 min from start to end for each participant enrolled in our study (n = 8).

#### Hemorrhagic stroke

**Figure 4:**
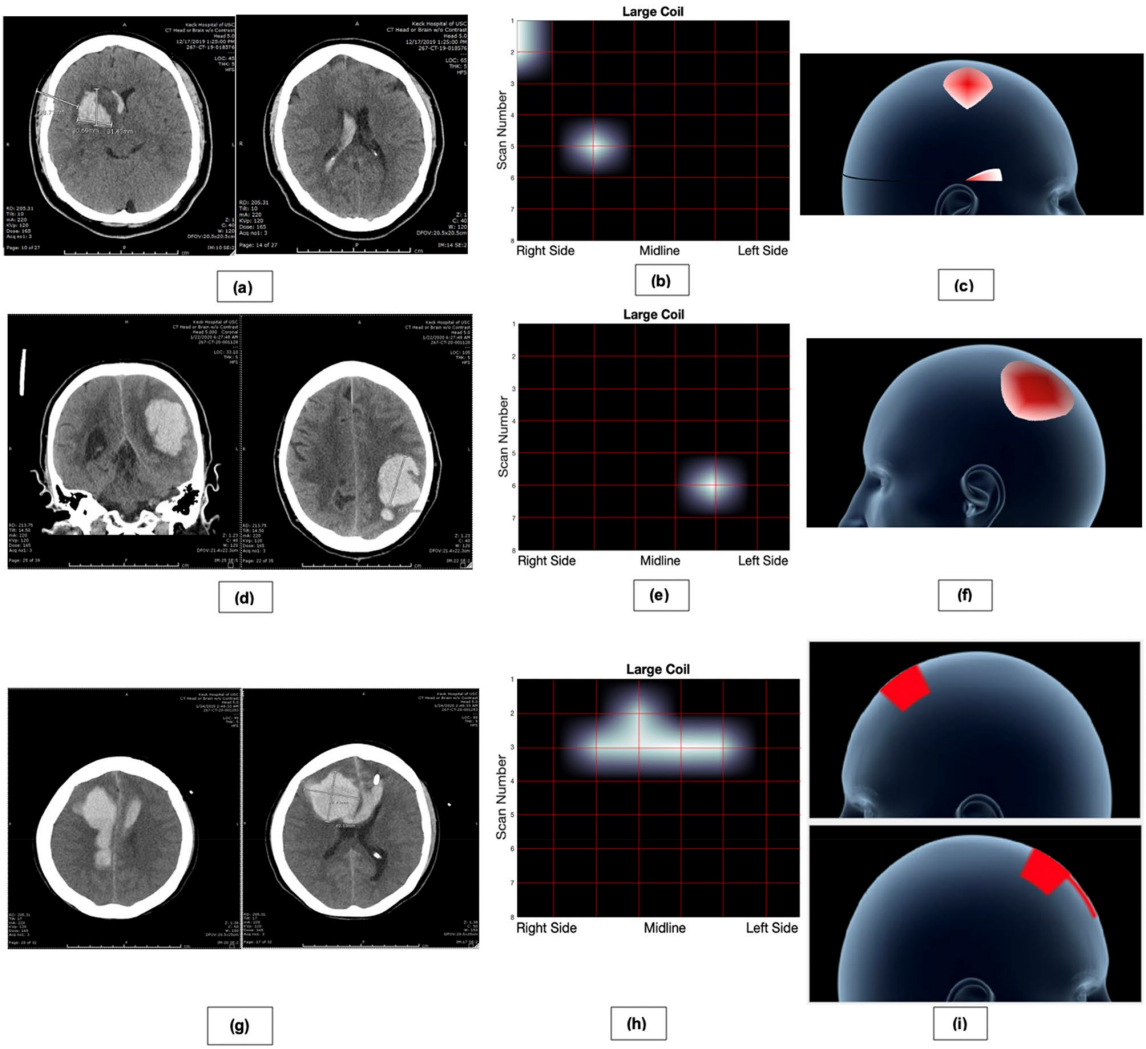
CT imaging, 2D ECD sensor imaging, and 3D ECD sensor imaging for three hemorrhagic stroke patients. The 2D images were produced by continuously scanning across the 8 scanning rows and then averaging each row into 8 equidistant points, with interpolation, to create an 8 × 8 heatmap. As such, lesions are shown on the vertices of the heatmap if detected. To create the 3D images, the 2D heatmaps were projected onto a hemispherical head template. (**a**) CT imaging showing a right basal ganglia ICH and associated IVH in Patient #1. (**b**) Two-dimensional data gathered from ECD sensor. Of note, the Large Coil has the largest magnetic field depth, and thus was able to detect both hemorrhages. (**c**) Three-dimensional projections of each coil. 3D graphics can be created in real-time to rapidly guide clinical judgement and reduce time-to-treatment. (**d**) CT imaging showing a left parietal lobar ICH in Patient #2. (**e**) Two-dimensional data gathered from ECD sensor scanning. (**f**) Three-dimensional projections of the lesion. (**g**) CT imaging showing bilateral ICH, right greater than left in Patient #3. (**h**) Two-dimensional data gathered from ECD sensor scanning. (**i**) Three-dimensional projections of the lesion. The lesion crosses the midline, so two images are provided with left (top) and right (bottom) profiles.

Patient #1 was a 32-year-old woman presenting with left-sided hemiparesis and placed on stroke protocol. CT imaging showed a 15 cc lesion in the right basal ganglia with the presence of moderate right intraventricular hemorrhage (IVH) (Fig. 4a). All coils were all used to collect data from the patient at bedside following CT imaging and all necessary stroke interventions. Both the 2D (Fig. 4b) and 3D (Fig. 4c) images produced by the sensor corroborated the presence of a hemorrhage in the right frontal lobe of the patient, and the range of the large coil (4.97 cm) allowed it to detect the right IVH. Both the small and medium coils were unable to detect the IVH likely due to their shorter scanning ranges, demonstrating their ability to provide additional depth information (Supplementary Fig. S1). By interpreting sensor outputs, we predicted that the IVH was between 4.97 cm and 3.99 cm from the surface of the skull, which was the confirmed depth range of the IVH at the right occipital horn of the lateral ventricle.

Patient #2 was a 78-year-old man who presented with right hemiparesis and loss of consciousness. CT imaging showed an 80 cc left parietal lobar hemorrhage. Scanning with the ECD sensor was performed, and image production corroborated the findings of the CT image (Fig. 4d–f, Supplementary Fig. S2).

Patient #3 was a 71-year-old woman with sudden onset loss of consciousness and coma. CT imaging revealed severe bifrontal, right greater than left ICH and subarachnoid hemorrhage (SAH) due to an aneurysmal rupture. Scanning with the ECD sensor revealed a diffuse hematoma, with the largest region of bleeding located in the right frontal lobe (Fig. 4g–i, Supplementary Fig. S3). Due to the diffuse nature of her hemorrhage, the ECD sensor was able to best detect the bleed at the area of maximum hemorrhage, as seen in the 2D and 3D images generated from the large coil. Both small and medium coils were able to locate the hemorrhage near the area of maximum hemorrhage, but failed to locate smaller regions of bleeding due to limited scanning depth.

#### Ischemic stroke

**Figure 5:**
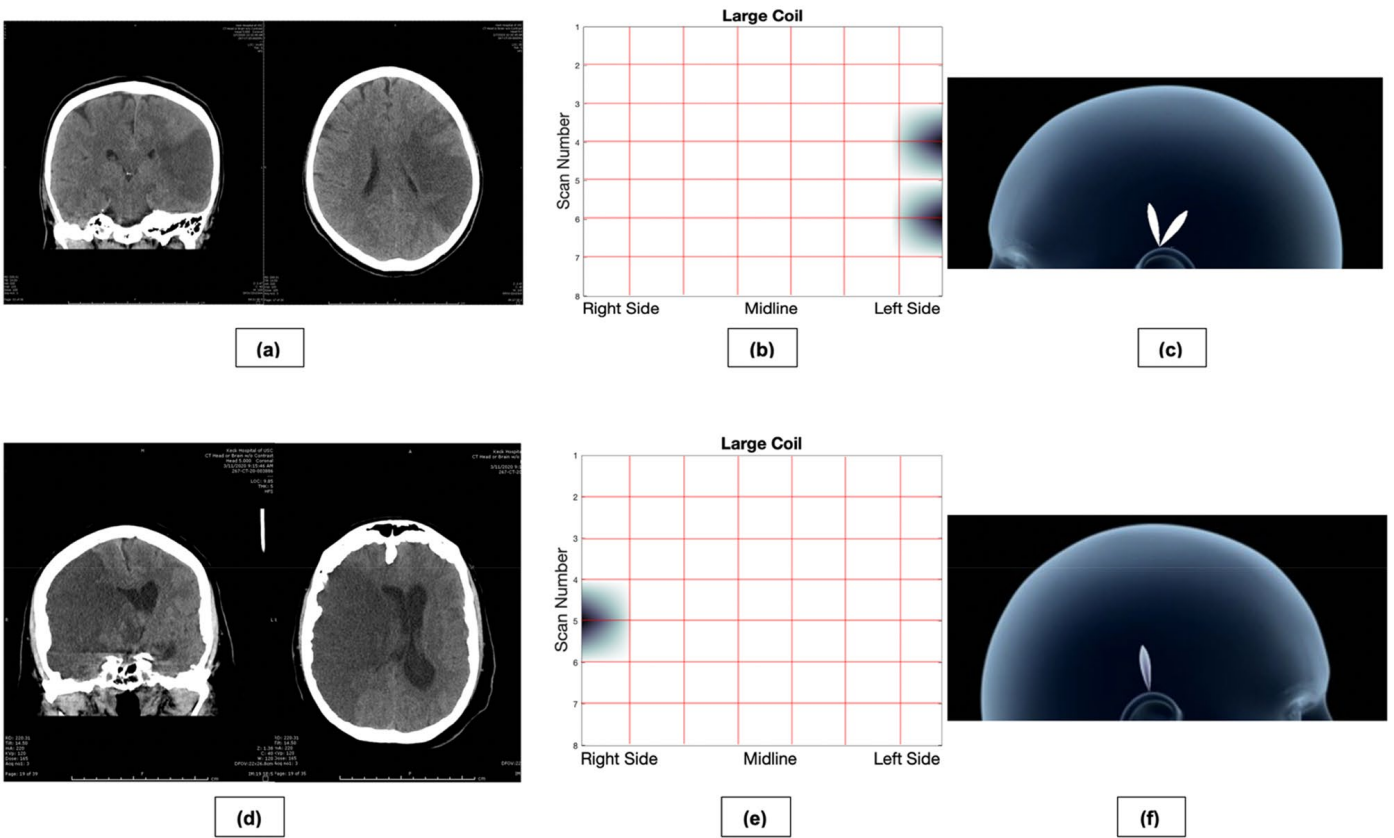
CT imaging, 2D ECD sensor imaging, and 3D ECD sensor imaging for two ischemic stroke patients. The 2D images were produced by continuously scanning across the 8 scanning rows and then averaging each row into 8 equidistant points, with interpolation, to create an 8 × 8 heatmap. As such, lesions are shown on the vertices of the heatmap if detected. To create the 3D images, the 2D heatmaps were projected onto a hemispherical head template. (**a**) CT imaging showing left MCA ischemic stroke. (**b**) Two-dimensional data gathered from ECD sensor scanning from Patient #4. (**c**) Three-dimensional projections of the maximal point of the lesion. (**d**) CT imaging showing right MCA ischemic stroke. (**e**) Two-dimensional data gathered from ECD sensor scanning from Patient #5. (**f**) Three-dimensional projections showing the maximal region of the ischemic lesion.

Patient #4 was a 50-year-old man with right arm weakness and aphasia, who presented with a left middle cerebral artery occlusion, resulting in developed regions of infarct and edema on CT imaging (Fig. 5a–c, Supplementary Fig. S4). The ECD sensor successfully localized the area of infarct to the left temporal and frontal region.

Patient #5 was a 77-year-old man with left hemiparesis and a large right MCA territory infarct on CT imaging. Scanning with the ECD provided localization of the lesion, shown in (Fig. 5d–f, Supplementary Fig. S5).

### Larger coils: experimental data and modeling

**Figure 6:**
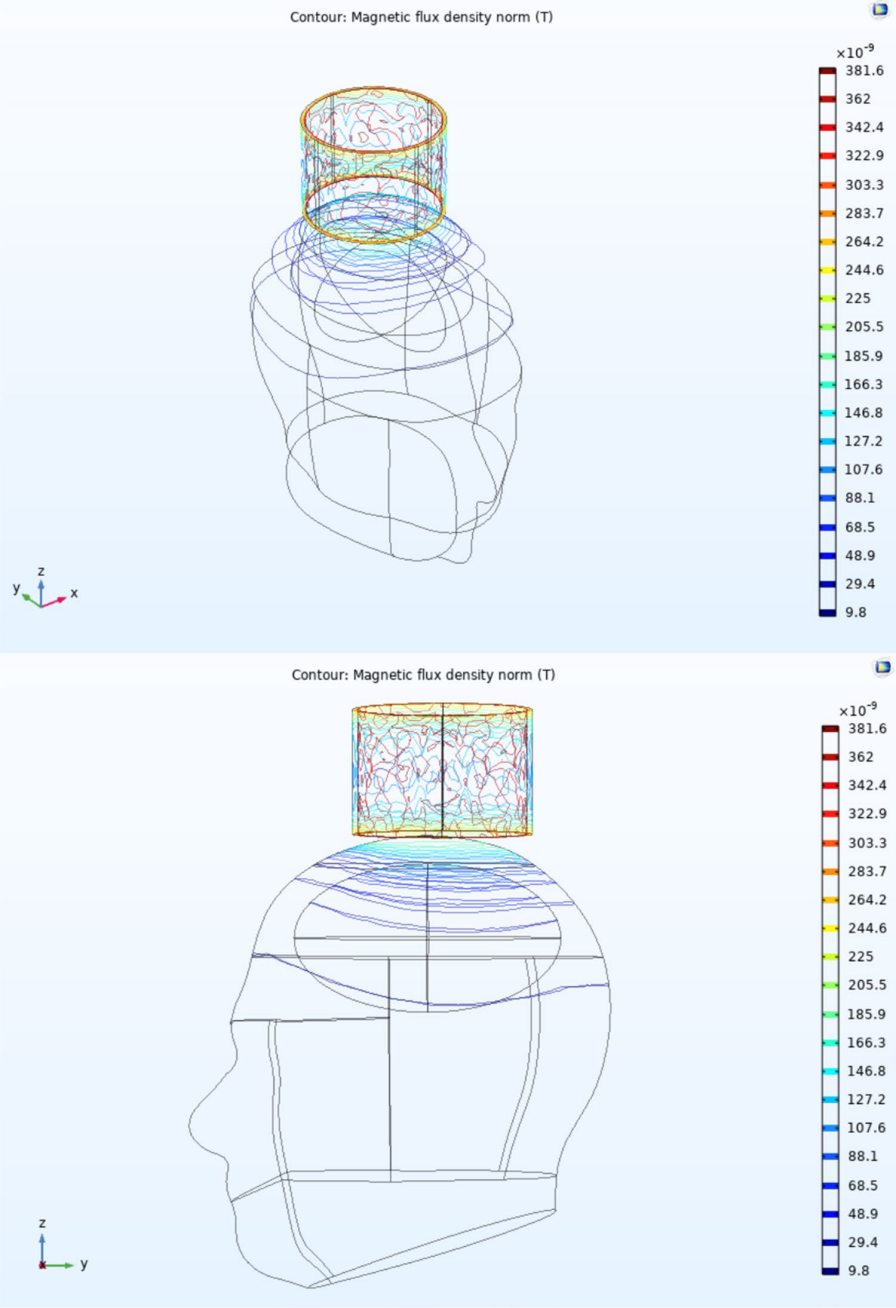
The 3D model of a human head with a coil sensor generated in COMSOL Multiphysics. The ellipsoid within the head is modeled as a lesion. As seen in both perspectives, the point of maximal lesion volume perpendicular to the coil, which also happens to have the closest proximity to the coil, produced the largest magnetic flux changes.

**Figure 7:**
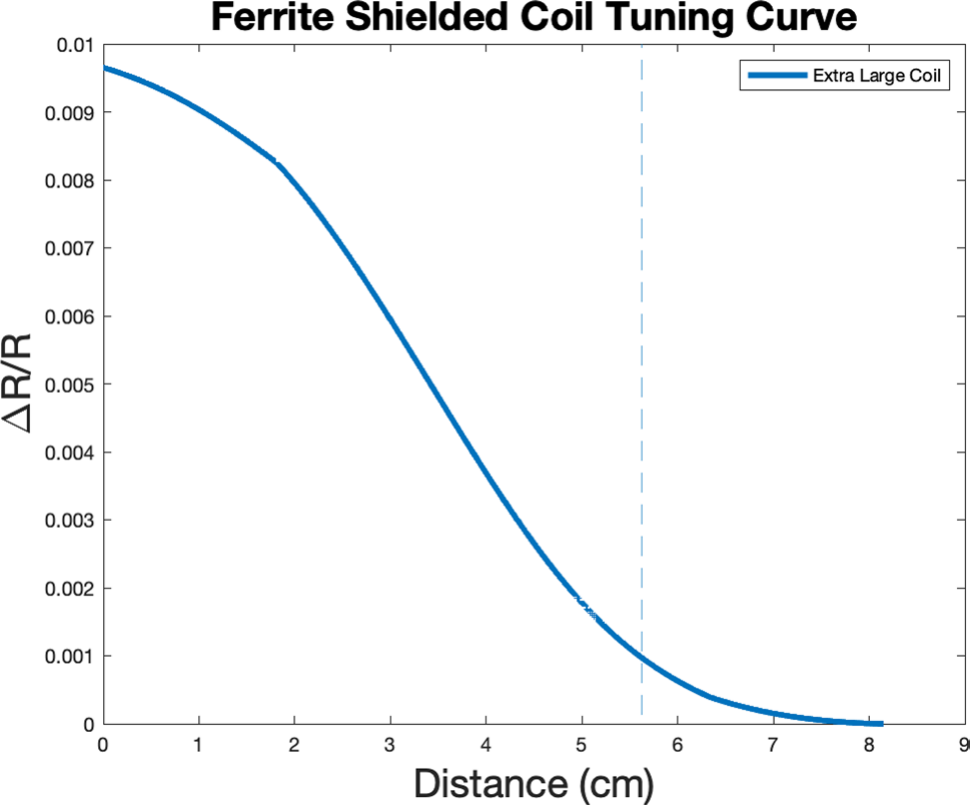
The extra-large coil (24 cm) was explored to confirm that detection depth increases with larger coil implementation. The curve above was produced by moving a 5 cm spherical balloon towards the extra-large coil at a constant rate, starting at a distance of 8 cm and moving closer until the balloon and sensor were touching (0 cm). Maximum sensor range was defined as a 10% change in resistance from baseline ($\frac{\mathrm{\Delta R}}{\mathrm{R}}$=0), which was determined to be the change in signal necessary to overcome noise (SNR = 10). At the point where the signal is 10% of the maximum generated signal, a vertical line is drawn and labeled the distance threshold (the point at which noise is overcome as defined by the SNR, and suggests confidence on signal detection). This coil was found to have a detection depth of 5.63 cm, which is larger than the scanning depths of the large, medium, and small coils described.

Although both infarcts occupy large cortical stroke territories in both patients, the images produced from the ECD sensor highlight the area of maximal ischemia, which in both cases was in the temporal region, ipsilateral to the lesion. To confirm this phenomenon, finite element modeling (FEM) was utilized to generate a three-dimensional model of the head (Fig. 6). The magnetic flux density generated when the coil was placed near the head showed the greatest strength in the area most proximal to the sensor’s center (Supplementary Fig. 6b). As such, the large magnetic flux density seen at the point of maximal ischemia may result in localization of a single point when aligned with the center of the sensor, corresponding to the location of the strongest magnetic field generated. In addition, larger coil sizes (24 cm diameter) were investigated using FEM simulations (Supplementary Figs. S6, S7). These additional FEMs demonstrated that extra-large coil sizes may scan deeper into brain parenchyma but may have more surface area interactions with the skull surface due to their circumferential design. To fully confirm this finding, additional tuning curve experiments were conducted (Fig. 7), and it was confirmed that the extra-large coil produced larger magnetic fields capable of scanning deeper into the brain (5.63 cm). Optimization of coil sizes and multiplexing may be explored as a future avenue to overcome the scanning depth and volume limitations of the current ECD sensor.

## Discussion

With stroke currently costing the US more than $71 billion per year, it is important to understand how we can make stroke diagnosis and treatment more efficient to minimize stroke morbidity and mortality^17^. The goal of this project is to develop a novel early stroke diagnostic device that can detect ischemic and hemorrhagic stroke, localize lesion location, and provide an image of the lesion to facilitate early treatment. Because the device is small and portable, it allows for rapid field and/or bedside diagnostics and reduced time to diagnosis and treatment, with hopes of reducing dependency on CT or MR imaging in rural or first responder settings and thereby lowering the morbidity and mortality associated with stroke. By providing the emergency care team with a rapid assessment of stroke status and subtype, it may also facilitate faster triage and tPA administration to augment clinical decision making and care.

Previous studies have described the disadvantages of traditional stroke imaging methods^3,4,18^. Namely, newer imaging procedures boast increased spatial resolution with a hefty time cost and financial burden^18^. Furthermore, although head CT imaging produces only small amounts of radiation, exposure to radiation through CT scanning has been linked to an increased rate of cancer over long time periods^19–21^. The ECD sensors used here scanned, imaged, and localized hematomas in minutes, with centimeter resolution. Additionally, the material used to create the ECD sensor costs < $100 for three coils, which is negligible compared to prices of diagnostic imaging machines which run in the multi-million-dollar range. Imaging machines also have operating costs, which are minimal in our device. Further, the novel sensor we have developed does not emit any ionizing radiation, thereby providing additional safety to patients. These results strengthen the role of ECD sensors in stroke care to image the brain and provide physicians with valuable information to guide treatment while avoiding radiation exposure, waiting times, and operating costs.

Similar devices using near-infrared spectroscopy (NIRS), volumetric impedance phase shift spectroscopy (VIPS), and microwave-based scanning have been developed with the hope of facilitating stroke triage and diagnosis^22–25^. While some have received Food and Drug Administration (FDA) approval and others show promise with regards to diagnosis, neither are able to produce images of the brain lesion in real-time. Furthermore, NIRS technology is severely limited by the depth of penetration of infrared light, limiting scanning to the most superficial 2.5 cm of the brain. While our ECD sensor is currently limited at a scanning depth of roughly 5 cm, sensor tuning through the development of slightly larger coils would easily allow for an increased scanning depth or volume detection (Fig. 7). Additionally, recent advances in low-field MRI technology have broadened the possibility of portable neuroimaging^26^. Although the MRI device was much larger than the ECD sensor and had higher power consumption, it was capable of achieving millimeter scanning resolution, which allows for the exclusion of small hemorrhages when deciding whether to administer tPA^26^. Because the ECD sensor currently achieves centimeter resolution, further experimentation and optimization are necessary to confidently exclude millimeter and sub-millimeter hemorrhages when classifying stroke subtypes and deciding whether tPA use is indicated. To address this, the authors present experimental and modeling data demonstrating increased scanning depths with large coil sizes, an avenue that may be explored to develop multiplexed ECD sensors with more than 3 coils that may be able to better resolve small hemorrhages and infarcts. While the ECD sensor was able to detect a 15 cc basal ganglia ICH and a smaller (~ 10 cc) IVH at the occipital horn of the lateral ventricle, further experiments investigating volume sensitivity are also necessary. In addition, another major advantage of the resonant circuit is the low power consumption, which is of great importance for portable and wearable sensors. Generating magnetic fields in the microtesla range requires very little power, allowing for device miniaturization and compactness for use in crowded ambulances and emergency rooms.

Although we demonstrate the use of an ECD sensor for pre-hospital triage of stroke patients, we also see a role of this device in rural and global medicine. It has been well studied that patients living in rural areas have less access to brain imaging modalities, resulting in increased stroke morbidity and mortality^27^. In addition, the rates of stroke morbidity and mortality in second and third-world countries surpass those of the US by a factor of ten^28^. Lack of access to modern imaging technologies and medical care results in astounding rates of stroke-related disability in the worldwide community^28,29^. Devices that are portable, cheap and easy to use might play a large role in addressing such health disparities.

### Limitations

Limitations of this work include the requirement for the ECD sensor to be isolated from metallic objects and magnetic fields when scanning and a small clinical patient series. However, this study is a feasibility study for a portable stroke sensor, which shows robust evidence of the capacity of an ECD sensor to detect ICH and differentiate it from its ischemic stroke counterpart. In addition, while higher sensitivity for hemorrhage works in favor for the clinical care thus facilitating administration of tPA, the lower sensitivity for ischemic stroke might result in confusing transient ischemic attacks with ischemic stroke, resulting in suboptimal decision of giving tPA. Lastly, while the coils described in this study show feasibility for stroke detection, further advancements regarding coil design and circuit architecture may further improve scanning abilities. By optimizing scaning depth and sensitivity, it may be possible to more accurately charactarize stroke using the sensor. Future work with a larger patient series aims at utilizing machine learning classification algorithms to classify stroke subtype and severity in real time. By doing so, such a device may provide information regarding both diagnosis and management concurrently to first responders or emergency physicians with hopes of facilitating patient care.

## Conclusions

We demonstrated feasibility of rapid and accurate bedside stroke detection using handheld ECD sensors in live human clinical ischemic and hemorrhagic stroke settings. We show that diagnosis of stroke may potentially be reduced from several hours to minutes, with additional spatial localization of intracranial hemorrhage or infarct. The sensor additionally detects ischemic and hemorrhagic lesions located deep inside the brain, and its range can be selectively tuned during sensor design and fabrication through the use of various sized coils. Further research is warranted to optimize the sensor for millimeter and sub-millimeter lesion detection to accurately and confidently guide clinical intervention.

## Supplementary Information

Supplementary Information.
