# Supplementary material for: Noninvasive transcranial classification of stroke using a portable eddy current damping sensor
Source: Sci Rep. 2021 May 13;11:10297. doi: 10.1038/s41598-021-89735-x (PMC8119677; doi:10.1038/s41598-021-89735-x)
Supplement: Supplementary file 1 — Supplementary Information. [file 41598_2021_89735_MOESM1_ESM.pdf]

Supplementary Materials Title Page

Title: Noninvasive Transcranial Classification of Stroke Using A Portable Eddy Current Damping Sensor

Authors: Shane Shahrestani MS[1,2], Gabriel Zada MD, MS[2], Tzu-Chieh Chou MS[1], Brandon Toy MS[1], Bryan Yao BS[1], Norman Garrett III BA[2], Nerses Sanossian MD[3], Andrew Brunswick MD[2], Kuang-Ming Shang MS[1], Yu-Chong Tai PhD[1]

Affiliations:

[1] Department of Medical Engineering, California Institute of Technology, Pasadena, California, USA.

[2] Department of Neurosurgery, Keck School of Medicine, University of Southern California, Los Angeles, California, USA.

[3] Department of Neurology, Keck School of Medicine, University of Southern California, Los Angeles, California, USA.

Supplemental Figures

/

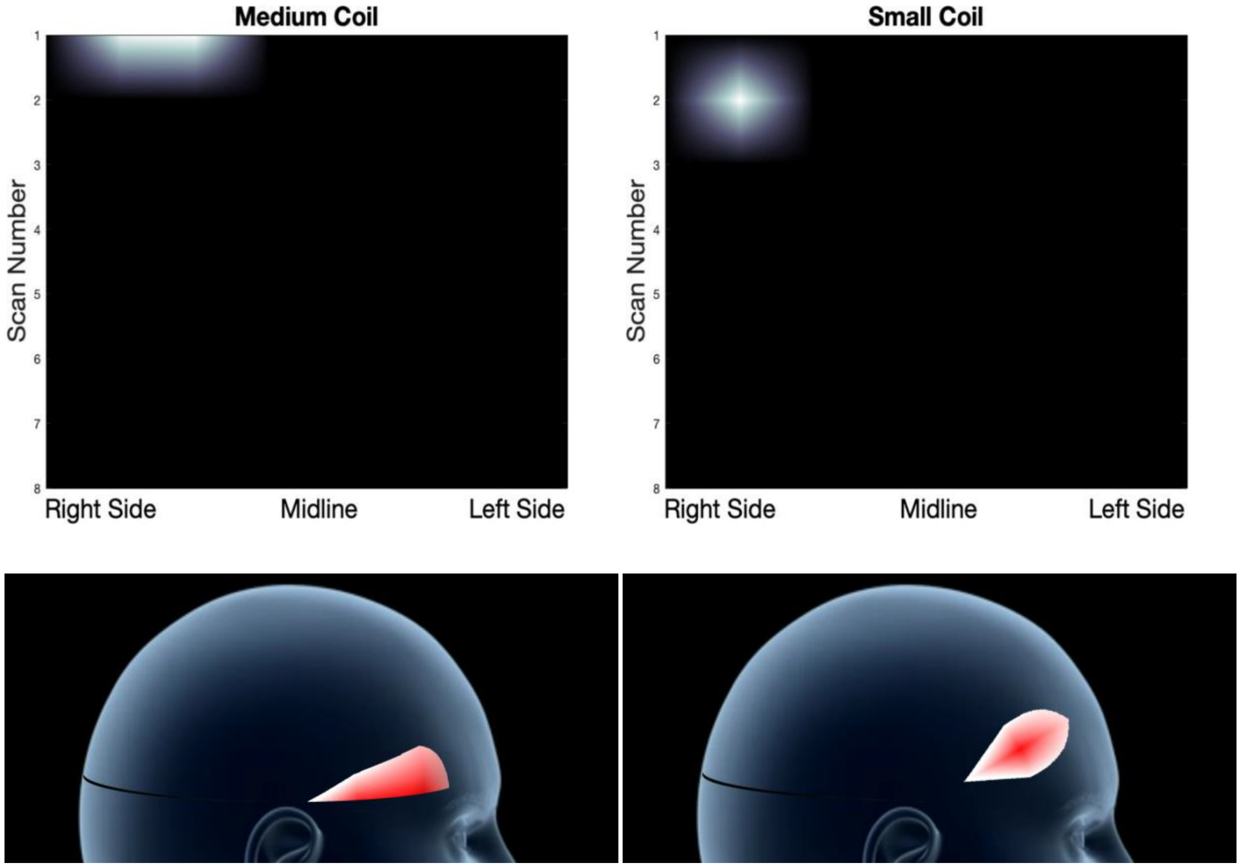

Supplementary Figure 1: *2D and 3D output for medium and small coils. While only the large coil is used for image production, the medium and small coils provide information regarding lesion depth, which can be seen in this example. While both coils pick up the larger basal ganglia hemorrhage, they both fail to detect the IVH. However, IVH was detected by the large coil with the deeper scanning depth.*

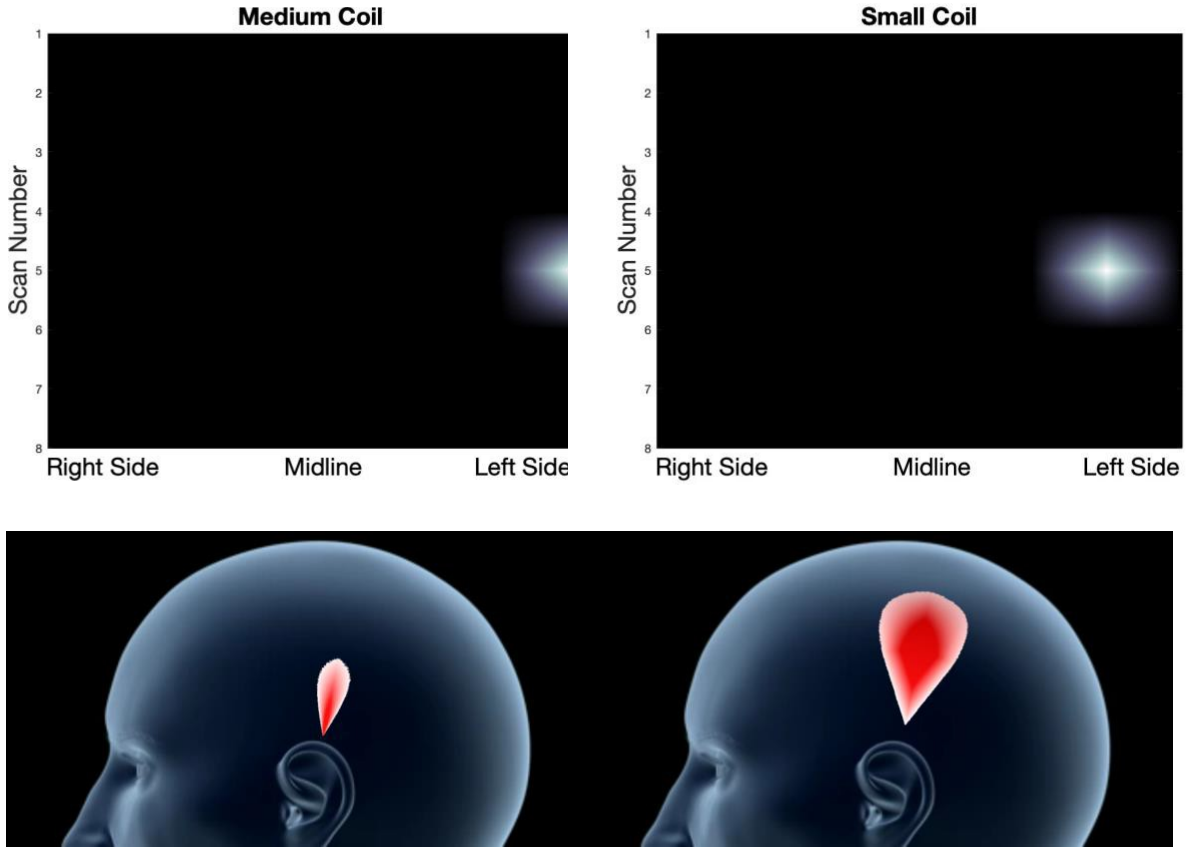

Supplementary Figure 2: *2D and 3D output for medium and small coils. The relative similarity between these scans and the large coil confirms that the bleed extends superficially and can be detected by all coils.*

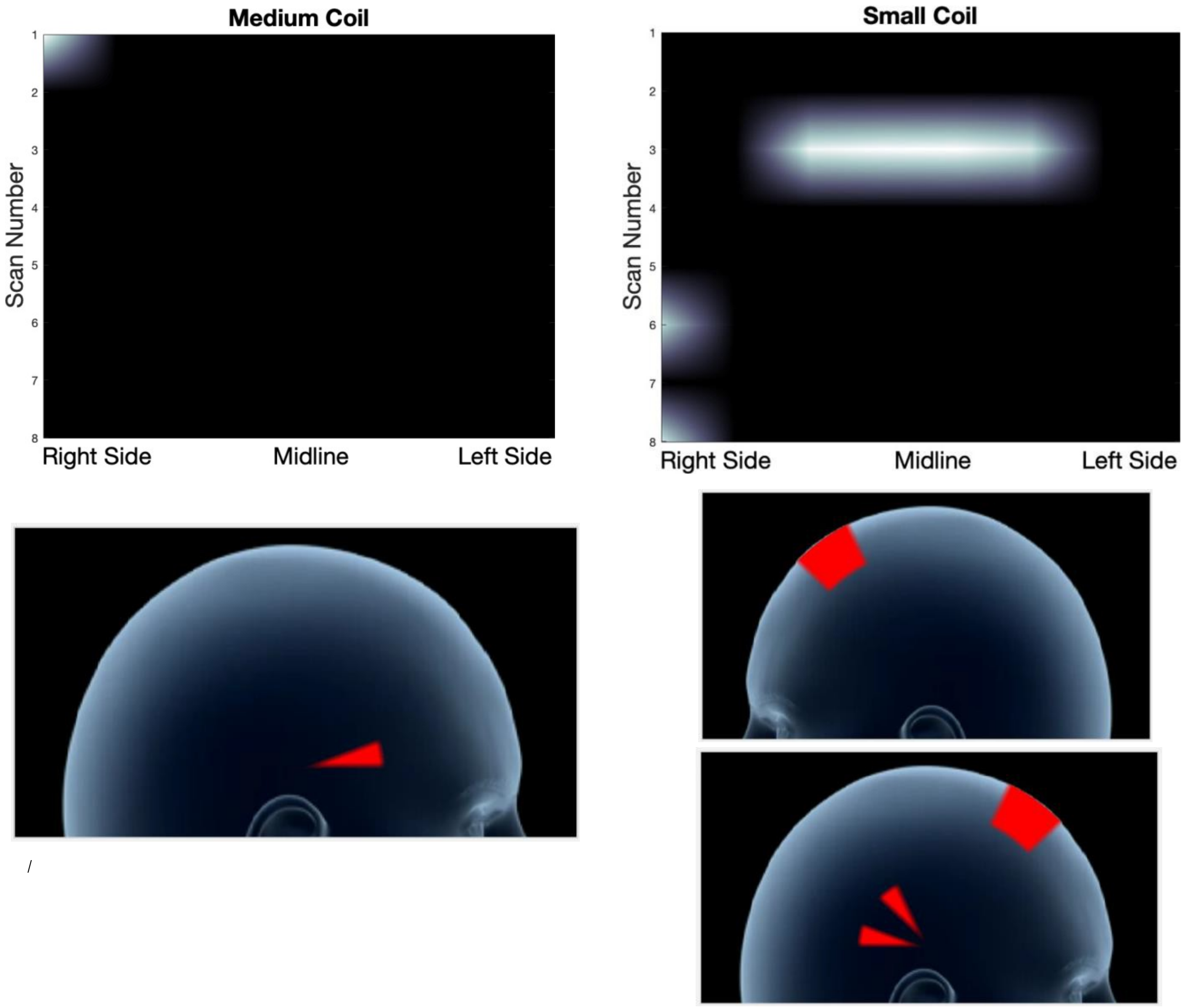

Supplementary Figure 3: *2D and 3D output for medium and small coils in the setting of a large SAH. Both coils (especially the medium coil) produce the largest signal at the point of maximal hemorrhage diameter or at points that extend superficially.*

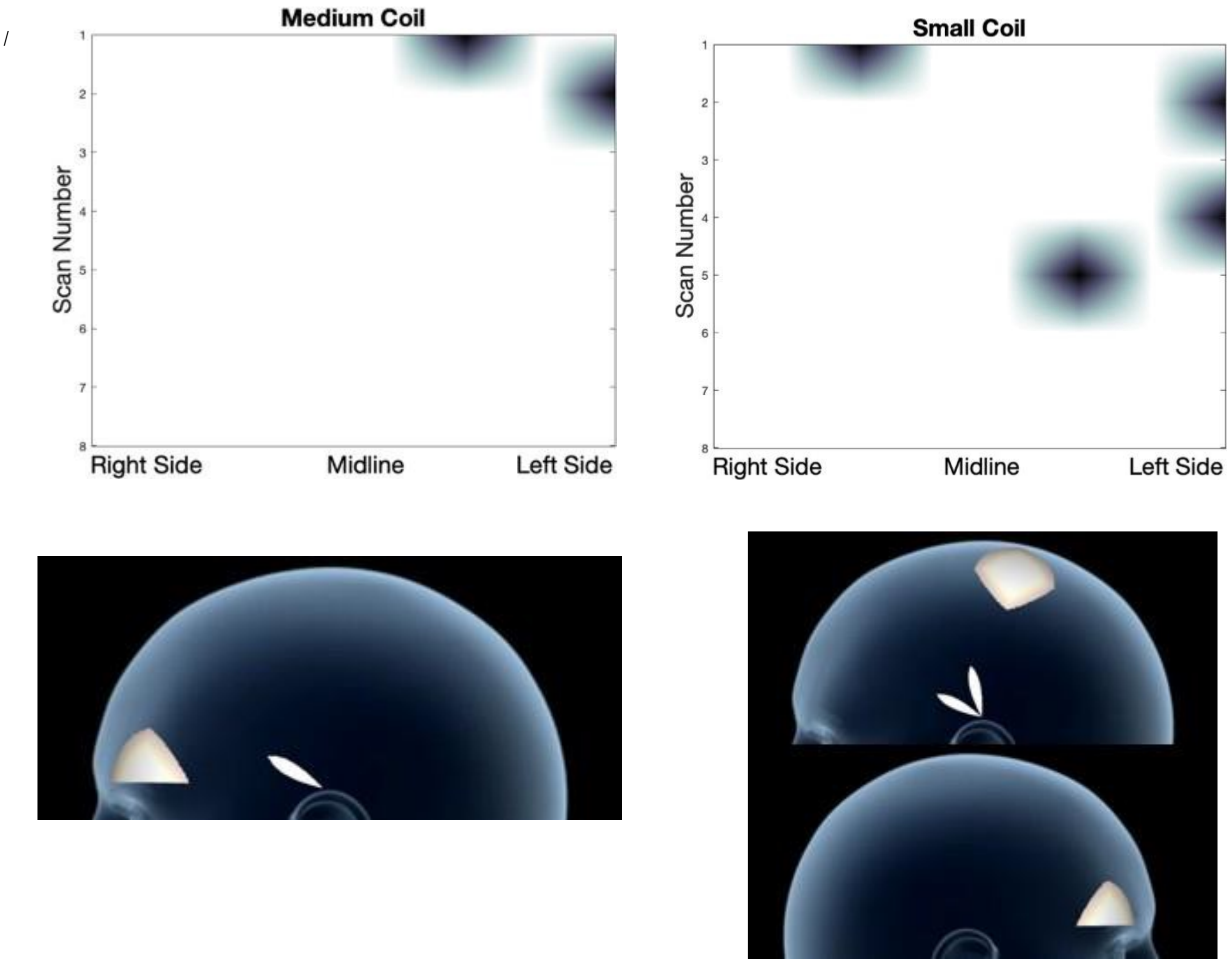

Supplementary Figure 4: *2D and 3D output for medium and small coils in the setting of a left MCA occlusion. While the large coil is more sensitive to areas of ischemia (hence why it is used for image production), the small and medium coils both suggest that the left MCA might be involved per the signal generated above the left ear.*

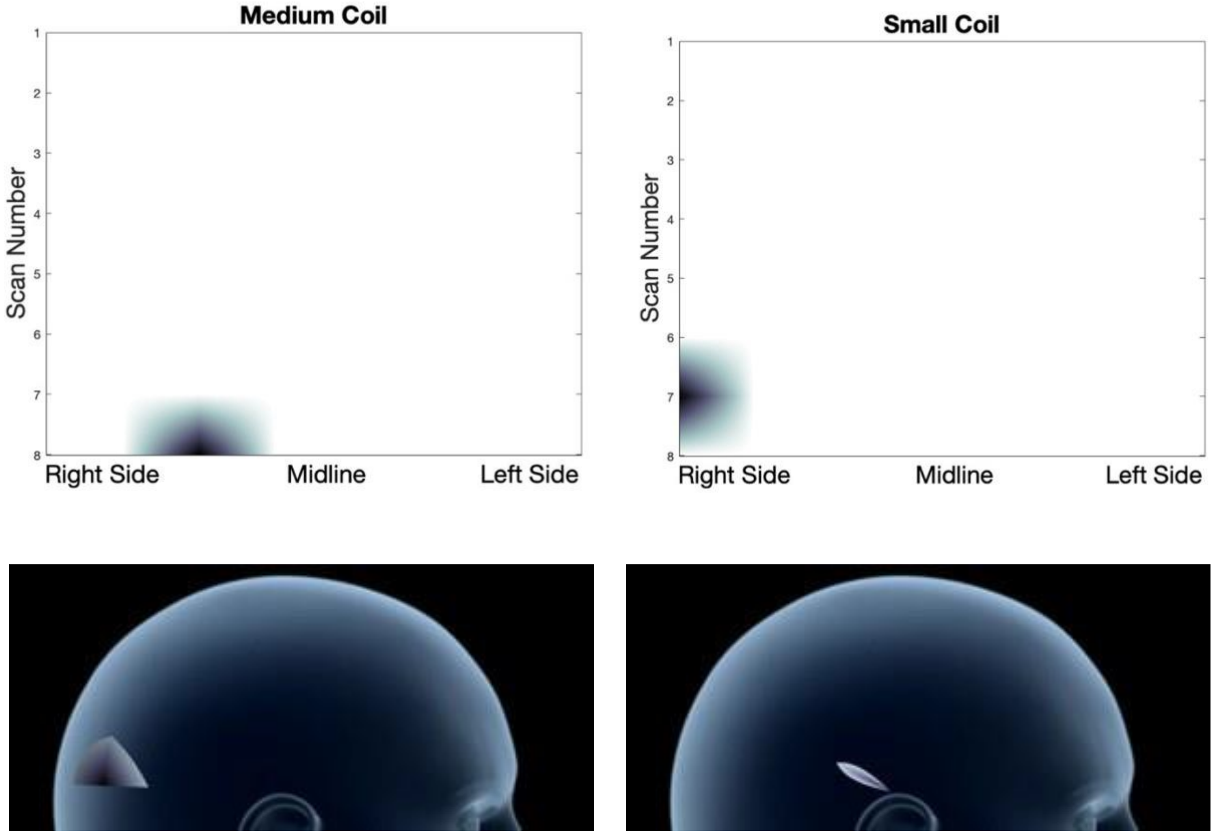

Supplementary Figure 5: *2D and 3D output for medium and small coils in the setting of a right MCA occlusion. While the large coil is more sensitive to areas of ischemia (hence why it is used for image production), the small and medium coils both suggest that there might be areas of ischemia on the right side of the patient.*

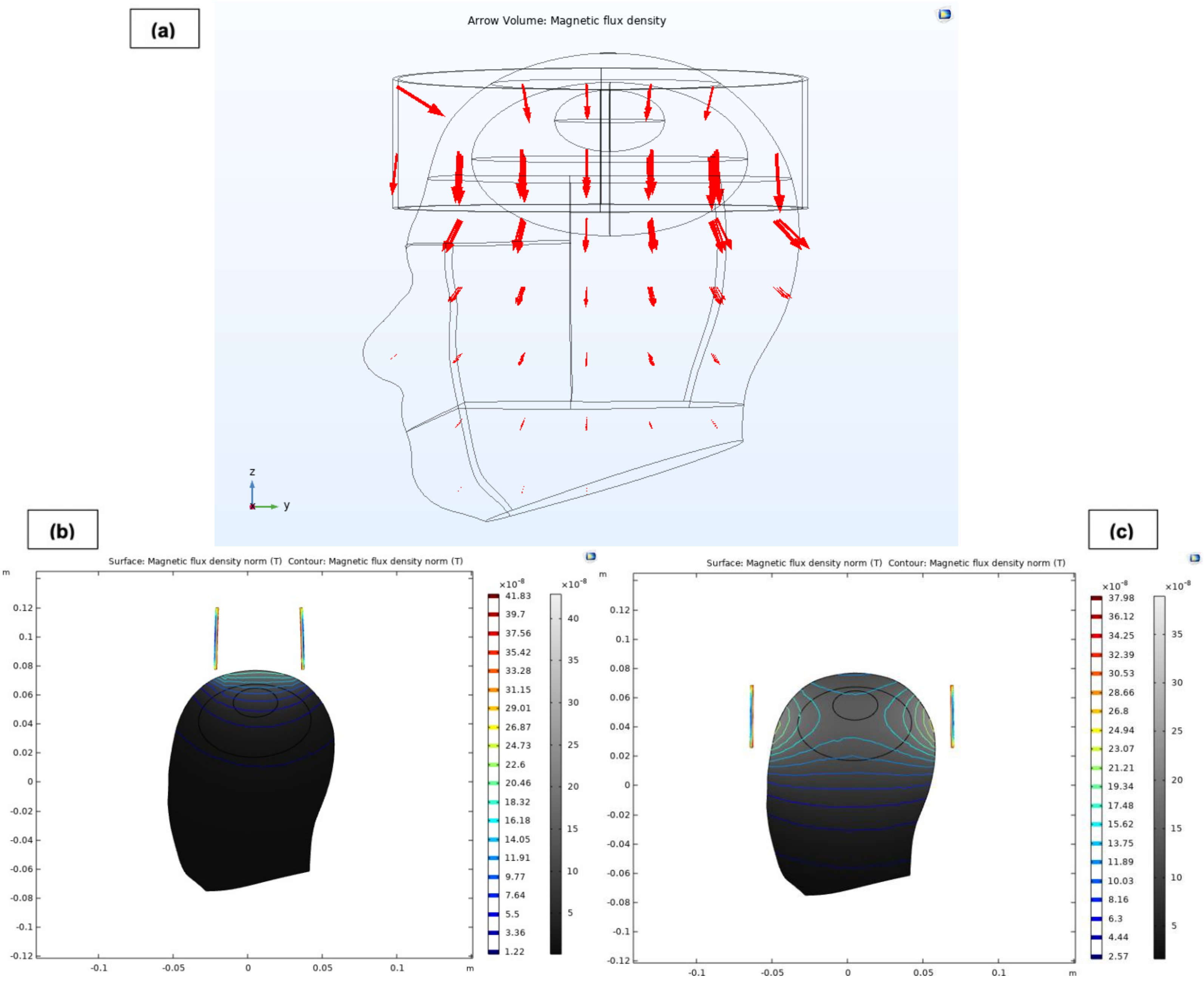

Supplementary Figure 6: *a) 3D finite element model of a 24cm coil with appropriate magnetic flux densities shown as arrows. b) 2D model of the 11.4cm large coil described in the study. c) 2D model of the 24cm coil demonstrating deeper scanning capabilities into the head.*

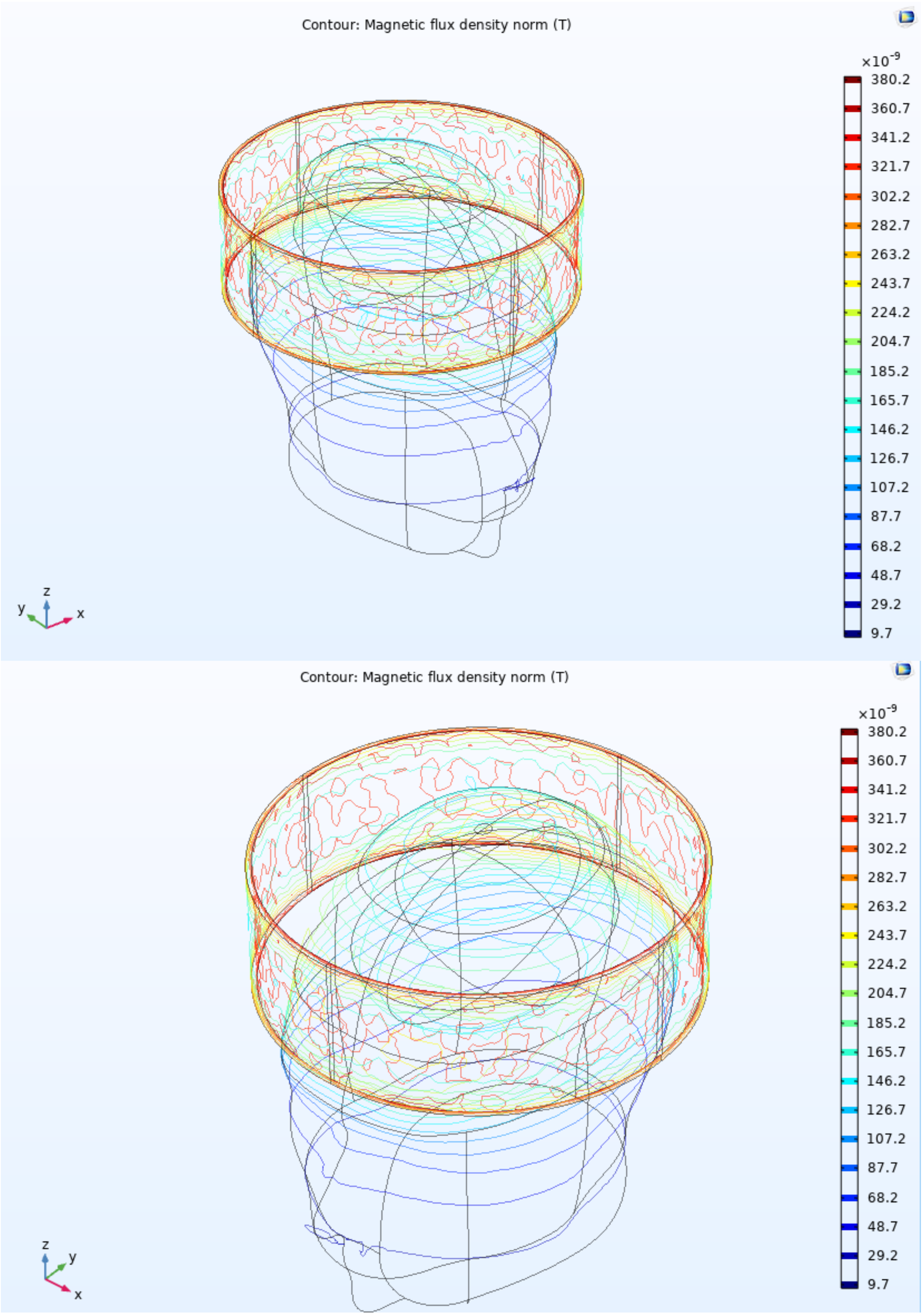

Supplementary Figure 7: *3D model of a human head with a coil sensor generated in COMSOL Multiphysics. The ellipsoid within the head is modeled as a lesion. Here, the feasibility of a larger coil (24cm) for deeper scanning within the brain is explored.*

Supplementary Equations

Biot-Savart's Law

$$B=\frac{\mu_0 N I r^2}{2(x^2+r^2)^{3/2}}$$

μ_0 = permeability of free space

N = number of turns

I = current intensity

r = radius

x = distance from center of the coil along axis of symmetry

Thus, we can calculate the expected magnetic field strength of each coil at a specific distance x:

Large Coil at x=0cm

$$B=\frac{4\pi*10^{-7}\frac{T*m}{A}*6*0.0027A*(0.057m)^2}{2([0m]^2+[0.057m]^2)^{3/2}} = 0.1786\ \mu T$$

Large Coil at x=5cm

$$B=\frac{4\pi*10^{-7}\frac{T*m}{A}*6*0.0027A*(0.057m)^2}{2([0.05m]^2+[0.057m]^2)^{3/2}} = 0.0759\ \mu T$$

Medium Coil at x=0cm

$$B=\frac{4\pi*10^{-7}\frac{T*m}{A}*15*0.0027A*(0.0225m)^2}{2([0m]^2+[0.0225m]^2)^{3/2}} = 1.131\ \mu T$$

Medium Coil at x=5cm

$$B=\frac{4\pi*10^{-7}\frac{T*m}{A}*15*0.0027A*(0.0225m)^2}{2([0.05m]^2+[0.0225m]^2)^{3/2}} = 0.0782\ \mu T$$

Small Coil at x=0cm

$$B=\frac{4\pi*10^{-7}\frac{T*m}{A}*35*0.0027A*(0.0075m)^2}{2([0m]^2+[0.0075m]^2)^{3/2}} = 7.917\ \mu T$$

Small Coil at x=5cm

$$B=\frac{4\pi*10^{-7}\frac{T*m}{A}*35*0.0027A*(0.0075m)^2}{2([0.05m]^2+[0.0075m]^2)^{3/2}} = 0.0258\ \mu T$$
